# Chemical Analysis of Whale Breath Volatiles: A Case Study for Non-Invasive Field Health Diagnostics of Marine Mammals

**DOI:** 10.3390/metabo4030790

**Published:** 2014-09-12

**Authors:** Raquel Cumeras, William H.K. Cheung, Frances Gulland, Dawn Goley, Cristina E. Davis

**Affiliations:** 1 Department of Mechanical and Aerospace Engineering, University of California, Davis, One Shields Avenue 95616, CA, USA; E-Mails: rcumeras@ucdavis.edu (R.C); hwcheung@ucdavis.edu (W.H.K.C.); 2 The Marine Mammal Center, 2000 Bunker Road, Fort Cronkhite, Sausalito 94965-2619, CA, USA; E-Mail: gullandf@tmmc.org; 3 Marine Mammal Education and Research Program, Marine Mammal Stranding Network, Humboldt State University, 1 Harpst Street, Arcata 95521, CA, USA; E-Mail: Patricia.Goley@humboldt.edu

**Keywords:** breath analysis, volatile organic compound (VOC), gas chromatography/mass spectrometry (GC/MS), marine mammal, gray whale

## Abstract

We explored the feasibility of collecting exhaled breath from a moribund gray whale (*Eschrichtius robustus*) for potential non-invasive health monitoring of marine mammals. Biogenic volatile organic compound (VOC) profiling is a relatively new field of research, in which the chemical composition of breath is used to non-invasively assess the health and physiological processes on-going within an animal or human. In this study, two telescopic sampling poles were designed and tested with the primary aim of collecting whale breath exhalations (WBEs). Once the WBEs were successfully collected, they were immediately transferred onto a stable matrix sorbent through a custom manifold system. A total of two large volume WBEs were successfully captured and pre-concentrated onto two Tenax^®^-TA traps (one exhalation per trap). The samples were then returned to the laboratory where they were analyzed using solid phase micro extraction (SPME) and gas chromatography/mass spectrometry (GC/MS). A total of 70 chemicals were identified (58 positively identified) in the whale breath samples. These chemicals were also matched against a database of VOCs found in humans, and 44% of chemicals found in the whale breath are also released by healthy humans. The exhaled gray whale breath showed a rich diversity of chemicals, indicating the analysis of whale breath exhalations is a promising new field of research.

## 1. Introduction

The chemical content of exhaled breath provides comprehensive information about the metabolic state of the subject, regardless of the species [1,2,3,4,5,6,7,8,9,10,11]. Studies on human breath have shown that it contains a mixture of volatile organic compounds (VOCs) in the gaseous phase [12], as well as non-volatile compounds that occur in small aerosolized droplets [13,14]. To date, 1840 biogenic VOCs have been identified in healthy humans [15]. Some of these are physiologically relevant indicators of health and disease, such as breath biomarkers indicative of specific bacterial, fungal, and viral respiratory infections [16,17,18,19], while others are associated with various types of cancer [12,20,21,22,23,24,25,26]. Breath analysis can also detect the presence of ingested compounds [27], exposure to specific exogenous substances from the environment [28,29], and exposure to stressful events [30]. The potential application of these measures to cetacean conservation physiology studies could provide information about the state of their metabolism through non-invasive monitoring. These studies may also provide an interesting animal model for human metabolism by comparing common metabolic markers.

Large whales produce a “blow” or clouds of mist as they exhale at the surface. A whale breath exhalation (WBE) is a mixture of gasses and droplets of condensed respiratory vapor. Researchers have realized that blow may represent a valuable, entirely non-invasive, physiological sample that can be collected with relative ease [31]. Individual whales may blow several times during a single surfacing interval, and it is possible for boats to approach the whale closely during this time interval. Recent developments in human breath research [32,33,34] have accelerated interest in developing this novel method for cetaceans.

Some human breath-sampling methods have been successfully modified for cetaceans, and it is important to note that human breath studies have shown that the sampling method can have profound effects on the analytical results [35,36]. In humans, inert Tedlar^®^ bags are often used for collection of whole breath [37,38,39], while non-volatile components are often collected via cooling of exhaled breath to produce ‘exhaled breath condensate’ (EBC) [40,41]. Further, volatile compounds can be collected with vacuum canisters [42] or sorbent traps [35,43,44]. In one case, blow samples from cetaceans have been collected from small trained odontocetes in captivity by holding an inverted tube or other device directly over the animal’s blowhole [45]. Blow droplets have been collected from both humpbacks and North Atlantic right whales (NARWs) using a variety of sampling devices attached to long poles positioned over the blowholes. Pole-based samplers have included the following: nylon fabric suspended across a 15 cm ring or a plastic framework [46]; Petri dishes [47] and a remote-controlled helicopter have been used to collect blow droplets on Petri dishes attached to the helicopter skids [48]. These techniques vary in the sampling volume, sampling rates, and types of information captured. For instance, the nylon sampler is likely to capture non-volatile compounds and mucus. The Petri dishes culture microorganisms for potential infectious disease diagnostics, yet it is sometimes difficult to attribute the cultured microorganisms to the cetacean respiratory tract. However, together these studies demonstrate that routine blow droplet collection from large whales is feasible. To our knowledge, direct analysis of exhaled respiratory gasses and VOCs in cetacean breath has not been attempted in previous studies. No sampling technique has focused on monitoring cetacean health in the field.

Two gray whales (*Eschrichtius robustus*), a mother with her calf, traveled 7 km up the Klamath River (Northern California, USA) in June, 2011, during their northward journey from breeding ground in Baja California to Alaska. Different attempts to encourage the whales to return to the sea were attempted, which included a variety of methods including playing calls of killer whales. After three weeks, the calf made it back to the Pacific Ocean, while the mother whale remained in the river. It is believed the calf was mature enough (4.5 m) to survive its journey to Alaska. The mother whale (13.7 m and 36,000 kg) died on 16 August after beaching on a sandbar in the river. Necropsy results indicated that the adult female gray whale died from septicemia presumed secondary to skin infection [49].

**Figure 1:**
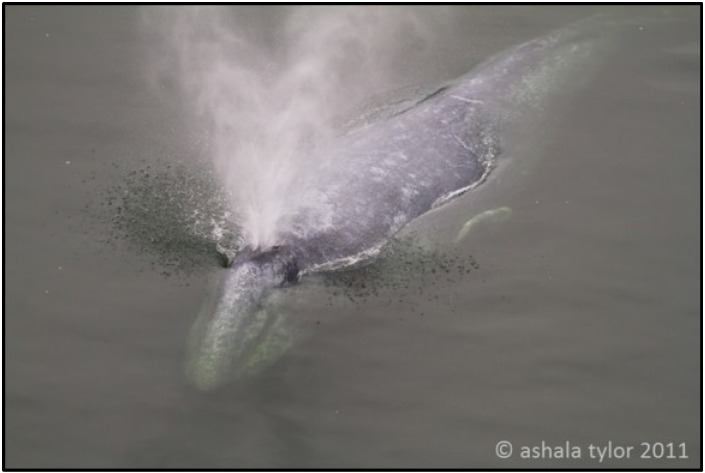
The adult female gray whale exhales respiratory vapor samples (“blow”) while swimming in the Klamath River. Printed with permission [50].

While researchers and wildlife officials were monitoring the whale in the river and attempting to return the female to the ocean, indirect measures such as measuring blow rate, movement patterns and collecting sequential photographs to document changing condition of the whale were being collected. The purpose of these measurements was not for the direct assessments of the whale’s health status. Our group was invited to collect breath for analysis from the animal, and a field trial was conducted between 31 July to 2 August, 2011, at the Klamath River Bridge, on Redwood Highway 101 (towards Crescent City, CA). The goal was to collect WBE samples from the adult female gray whale as non-invasively as possible and examine the feasibility of determining the whale breath metabolic content without direct contact with the animal. Figure 1 shows the exhaled blow of the mother gray whale while swimming in the Klamath River.

## 2. Methods and Materials

### 2.1. Fabrication of WBE Sample Collection System

**Figure 2:**
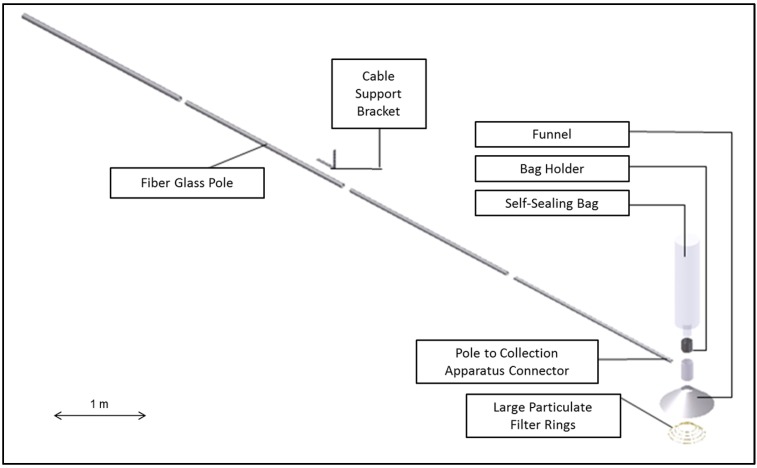
Schematic layout of the initial sampling pole showing the individual components.

A 7 m fiberglass sampling pole was designed and fabricated with a focus aperture to channel the WBEs into an inert large volume sampling bag intended to capture whale breath and contain it for further analysis (Figure 2). A 30 L capacity sample bag (Ingeniven LLC; Hampton, NH, UK), composed of Teflon^®^, was attached to one end of the pole. This was chosen to minimize potential background contamination. This bag material has significant inertness for short-term storage of the WBEs prior to transferring the breath chemicals onto a more stable sorbent matrix for ground transport and subsequent chemical analysis back in the laboratory. The materials also have the structural strength to withstand the force of rapid exhalations by the whale.

**Figure 3:**
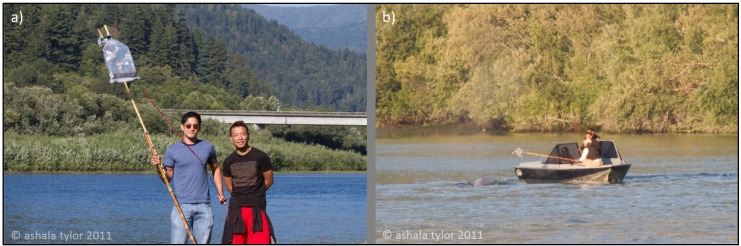
(**a**) Lighter bamboo sampling pole with the Teflon bag attach to one end. (**b**) Method used to collect breath from the gray whale in the Klamath River. Printed with permission [51].

The telescopic sampling pole was originally designed to collect WBEs from humpback whales in the open ocean by rotating the pole in a semi-stationary manner with an O-lock interface on the deck of a large research vessel. During our field test, we observed the fiberglass pole generated significant drag due to our vessel moving at a relatively moderate speed on the water. This combined with the relatively narrow channel of the Klamath River with shallow banks made the sampling procedure more challenging than anticipated. Modifications of our sampling approach were needed, and the fiberglass pole was determined to be unsuitable for our small boat without an O-lock interface for positioning and collection. A second lighter sampling pole was designed and fabricated from bamboo (Figure 3a). This pole could be easily positioned by only one person, and it had greater flexibility for use with the boat. The same sampling bag was used for both sampling poles (30 L) to enable the collection of as much sample volume from the whale’s exhalations as possible.

### 2.2. Field Sampling Methodology

Due to the field constraints of the Klamath River environment where the whale was located, a small single engine motor boat operated at low speed was used to sample the blow of the gray whale (Figure 3b). Exhaled breath samples were collected only when the calf was not nearby the mother gray whale, to ensure we did not interfere with their interactions. The sampling pole and collection bag were positioned vertically above the blow hole to collect the WBEs directly as the whale was exhaling. This proved to be challenging, as the gray whale was continuously moving. The experienced boat operator was able to reposition the boat to match the whale’s movements allowing the correct positioning of the sample collection equipment above the blowhole. Through this process, we successfully sampled two WBEs that were stored for off-site analysis of VOCs.

### 2.3. Stabilization of Breath Gasses for Ground Transport

Breath gas samples can be extremely unstable over time and the chemicals can easily degrade if not stored properly. They also potentially contain VOC signatures associated with the ambient environment. Once a breath sample was successfully collected, it was immediately transferred and pre-concentrated onto a hydrophobic Tenax^®^-TA sorbent trap and capped with Swagelock^TM^ fittings. This provided an extended duration when the samples were stable and could be transported with minimal contamination or degradation. The dominat component of whale exhaled breath is water and the effect of condensation and resulting potential loss will play a significant role if the sample remains in the bag for an extended amount of time. Individually pre-cleaned bags were used for each sample and were not reused afterward to minimiz artifact effects. In addition, field sample blanks were also collected and pre-concentrated onto a separate Tenax^®^-TA trap before the WBEs were collected. Sample blanks are needed for assessment of potential background chemical levels present in the ambient air during the field sampling. Direct SPME infield sampling attached to the pole was not considered as we could not ensure the cleanliness of the SPME fiber upon storage, transport and sampling. Therefore, we decided to attach a Teflon^®^ bag to the sample pole and immediately transfer the obtained exhaled breath to a hydrophobic Tenax^®^-TA sorbent to collect the breath biomarkers for short time shortage and transport. Furthermore, the Tenax-TA sorbent is more suited for the trapping of volatile compounds rather than the non-volatile components of breath profiles.

Each Tenax^®^-TA trap was housed in a quartz wide bore gas chromatograph (GC) liner containing 200 mg (±4 mg) of Tenax^®^-TA sorbent (mesh size 60/80, Sigma Aldrich, St. Louis, MO, USA). The traps were conditioned in replicates (n = 3) using a custom machined aluminum heating block with Swagelock^TM^ connections. The manifold was purged with nitrogen at rate of 50 mL/min at 300 °C for 6 h. This conditioning process within inert atmosphere purged residual compounds remaining on the sorbent, and provided a “blank” substrate to trap biologically relevant exhaled breath compounds for analysis. Once conditioned, the traps were then allowed to cool for an additional hour prior to being removed and tightly capped with prebaked Swagelock^TM^ fittings and corresponding blanking nuts, similar to a commercial thermal desorption tube. A third of our conditioned Tenax^®^-TA traps were analyzed to check for artifacts and background levels of exogenous compounds.

To transfer the WBE sample into the trap, the inlet end of the GC liner was connected to a 200 mL/min flow regulator to meter the gas flow through the trap to ensure reproducibility. The typical transference time from the sampling to the sorbents is 20–30 min. Within the sampling, approximately 1/4 to 1/5 of the effective 30 L volume was successfully used for collection. The initial (60 s) portion of sampling was not collected and was used to purge the flow manifold. The end of the flow meter was then carefully inserted into the Teflon^®^ bag containing the whale’s breath. A tight seal was created on the exterior of the bag using multiple layers of PTFE tape and cable ties to ensure minimal whale breath gasses escaped from the bag during transfer. A flow regulator was connected such that constant pressure was applied to the sampling bag, forcing the WBE through the regulator and the GC liner. The breath was transferred directly onto the sorbent trap in the field for both WBE and air blanks. Then they were returned to the UC Davis campus for biochemical analysis.

### 2.4. Chemical Analysis of the WBE

SPME fibers of 75 µm thickness PDMS/CAR black coating (Sigma Aldrich, St. Louis, MO, USA) were used to introduce the breath gas sample into the gas chromatography/mass spectrometer (GC/MS). These fibers were conditioned at 300 °C for 1 h on the auto sampler heater block prior to being used, and were periodically analyzed to check for artifacts and contaminations. Each individual Tenax^®^-TA sorbent trap was transferred into a clean prebaked 2 mL borosilicate vial and capped. The vials were then purged with ultra-high purity nitrogen 99.9995% (Airgas; Sacramento, CA, USA) at 10 mL/min for 5 min to ensure all oxygen was excavated from the vial prior to heating in order minimize any uncontrolled oxidation reactions with whale breath metabolites that could be released from the sorbent. The vials were then placed onto a hot plate (pre-heated to 280 °C) for 3 min. The vials were subsequently removed from the plate, and one clean SPME fiber was inserted into the vial and left exposed for 15 min to collect any biogenic VOCs that thermally desorbed from the Tenax^®^-TA and onto the SPME fiber.

The SPMEs were analyzed with a Varian 4000 Gas Chromatograph / Mass Spectrometer (GC/MS) (Agilent; Santa Clara, CA, USA) with a CombiPAL auto sampler system (CTC Analytics; VA, USA) and a liquid nitrogen cooling system for low temperature GC/MS analysis. The analytical column was a Factor Four VF5ms 30 M × 0.25 mm with a film thickness of 0.25 µm. The GC profile was: initial temperature 0 °C hold for 20 min; then ramp at 1 °C/min to 75 °C (hold for 15 min); then ramp to 100 °C (hold for 15 min); then ramp at 5 °C/min to 125 °C (hold for 5 min); then ramp at 5 °C to 150 °C; and finally ramp at 5 °C/min to 200 °C.

### 2.5. Data Analysis and Interpretation

Data analysis and mass spectral interpretations were carried out on the instrument vendor software [52] and automated mass spectral de-convolution and identification system (AMDIS) with medium resolution and high sensitivity and shape requirements. MS matching was performed using NIST 05 databases. By comparing the field sample blank to the WBEs, any peaks that appear in the field sample blank and the WBE samples can be effectively disregarded, as they likely came from the background or from impurities introduced during the sample transfer steps rather than of anything of biological significance. Only peaks that appeared in the WBEs sample were considered.

## 3. Results and Discussion

**Figure 4:**
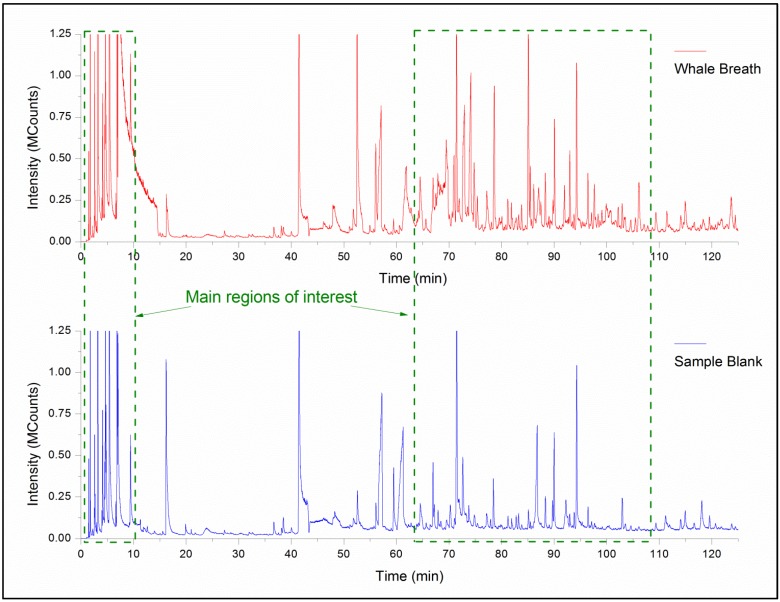
GC/MS chromatogram of the exhaled whale breath (top) and the sample blank (bottom). In addition, the main regions of interest (regions with most of the relevant VOC’s) are indicated.

The sampling procedure applied in the collections of WBEs demonstrate good signal intensity and high signal-to-noise ratio with over 150 chemicals observed in the raw GC/MS spectra. A chromatograph of the whale exhalation and an air blanks are shown (Figure 4). The background blanks were used to identify and remove contamination/artifacts. In total, 70 chemicals were identified as unique to the WBE. Of those chemicals, 58 were identified to come from the whale breath itself and from those 25 were successfully identified through mass spectral matching with NIST05 (compounds with + in Table 1). The peak IDs, retention times (RT), fragmentation patterns, potential matches and additional metadata are shown (Table 1). Forward match (match in Table 1) is the similarity of the fragmentation profile from low to high mass-to-charge ratio m/z values, whereas the reverse match score is the similarity of the fragmentation profile from high to low m/z values. The total match score is the average of the two. The CAS number and NIST ID are also provided in Table 1. Many of the compounds identified also match with biogenic VOCs that have been identified in healthy humans [15]. From the 70 identified compounds, 31 of them (44%) were found to be also present in healthy human volatiles.

**Table 1:** Compounds identified in the moribund gray whale breath by GC/MS. Reported are the peak number, retention time (RT), chemical name, CAS number, forward match (Match), reverse match (R. Match), relative abundance (RA), chemical formula, molecular weight (MW), the NIST identification number and the Human volatile organic compound (VOC) Reference when available: Feces (F), urine (U), breath (Br), skin secretions (Sk), milk (M), blood (Bl) and saliva (Sa). Compounds successfully identified based on spectral library match are shown with a + after the RT value.

RT (min)	Chemical Name	CAS	Match	R. Match	RA (%)	Formula	MW	NIST	Human VOC Ref.
1.477^+^	Carbon dioxide	124-38-9	682	696	2.32	CO_2_	44	18873	F, Br, Bl
1.549	Carbonyl sulfide	463-58-1	943	952	0.06	COS	60	19006	Br, M
2.592	Guanidine	113-00-8	811	831	4.98	CH_5_N_3_	59	221220	
2.668	Furan	110-00-9	758	767	0.59	C_4_H_4_O	68	228308	U, Br, M
2.960^+^	Cyclopropane, 1,1-dimethyl-	1630-94-0	899	899	0.21	C_5_H_10_	70	34618	
3.191^+^	Carbon disulfide	75-15-0	783	785	100.00	CS_2_	76	118705	F, U, Br, Sk, M
3.360	2-Propanol, 2-methyl-	75-65-0	796	809	0.34	C_4_H_10_O	74	19162	Br, M, Bl
3.999^+^	Butane, 2,3-dimethyl-	79-29-8	849	855	0.44	C_6_H_14_	86	291518	Br
4.129^+^	Octane	111-65-9	847	850	0.95	C_8_H_18_	114	229407	F, Br, Sk, Sa
4.507	2,3-Dihydrofuran	1191-99-7	783	849	4.37	C_4_H_6_O	70	114595	F, Br
4.635	Pentane, 2,3-dimethyl-	565-59-3	756	757	4.15	C_7_H_16_	100	61339	Br
6.643^+^	Pentane, 2,2-dimethyl-	590-35-2	852	876	12.01	C_7_H_16_	100	114486	Br
6.867^+^	2,4,6-Trimethyl-3-heptene	126690-66-2	721	765	1.51	C_10_H_20_	140	113569	
9.417	non-ID				15.60				
15.017^+^	Methane, dibromo-	74-95-3	928	928	3.64	CH_2_Br_2_	172	118720	Bl
27.312^+^	Toluene	108-88-3	917	917	0.00	C_7_H_8_	92	227551	F, U, Br, Sk, M, Bl, Sa
31.912	2-Butene, 2-methyl-	513-35-9	808	808	0.47	C_5_H_10_	70	242	Br
36.689	non-ID				0.15				
47.926^+^	o-Xylene	95-47-6	879	879	0.49	C_8_H_10_	106	228061	F, Br, M, Bl, Sa
51.064	non-ID				0.84				
51.844^+^	Ethylbenzene	100-41-4	670	798	0.14	C_8_H_10_	106	114918	F, U, Br, Sk, M, Bl
54.970	Decane	124-18-5	816	816	1.04	C_10_H_22_	142	227682	F, Br, Sk, Sa
56.087^+^	Ethanol, 2-butoxy-	111-76-2	834	834	0.39	C_6_H_14_O_2_	118	11812	U, Br
57.765	non-ID				12.19				
64.212	Benzene, (1-methylethyl)-	98-82-8	920	920	0.05	C_9_H_12_	120	114201	F, Br
64.494^+^	Ethanone, 2,2-dihydroxy-1-phenyl-	1075-06-5	874	894	0.18	C_8_H_8_O_3_	152	289212	F
65.635	Benzene, 1,2,3-trimethyl-	526-73-8	719	843	7.33	C_9_H_12_	120	125395	F, Br, M
70.597^+^	Indane	496-11-7	618	780	0.63	C_9_H_10_	118	20399	F, Br
70.931	3-Aminopyridine	462-08-8	671	692	0.06	C_5_H_6_N_2_	94	288371	Sk
72.982	non-ID				6.15				
73.547	Benzene, 1-ethyl-4-methyl-	622-96-8	772	827	1.19	C_9_H_12_	120	114034	F, Br, M
74.159^+^	Butane, 2-methoxy-2-methyl-	994-05-8	690	700	0.87	C_6_H_14_O	102	61443	
74.776^+^	Benzene, 1-methyl-4-(1-methylethyl)-	99-87-6	911	920	9.71	C_10_H_14_	134	113988	U, Br, Sk, Sa
75.411^+^	Limonene	138-86-3	764	805	6.13	C_10_H_16_	136	57640	F, U, Br, M, Sa
77.196	1-Hexene, 3,5-dimethyl-	7423-69-0	630	739	2.04	C_8_H_16_	112	113470	
77.392^+^	Benzyl Alcohol	100-51-6	827	891	3.18	C_7_H_8_O	108	291348	F, U, Br, Sk, Sa
78.631	1-Butanol, 4-(1-methylethoxy)-	31600-69-8	768	800	0.20	C_7_H_16_O_2_	132	46521	
78.888	Benzene, 1-methyl-3-propyl-	1074-43-7	573	781	5.70	C_10_H_14_	134	57774	
79.357^+^	2(3H)-Furanone, dihydro-4-methyl-	1679-49-8	772	833	0.60	C_5_H_8_O_2_	100	153216	
80.062	non-ID				0.62				
81.189^+^	Benzoyl bromide	618-32-6	916	916	0.43	C_7_H_5_BrO	184	228984	
81.875	2-Butene-1,4-diol, diformate	29619-56-5	809	818	3.27	C_6_H_8_O_4_	144	4155	
82.071	Hexanoic acid, 2-tetrahydrofurylmethyl ester	2217-34-7	684	772	3.03	C_11_H_20_O_3_	200	279279	
82.594^+^	Benzene, 2-ethyl-1,3-dimethyl-	2870-04-4	825	830	0.73	C_10_H_14_	134	3144	
84.652	non-ID				0.30				
85.086^+^	Benzene, (2-methyl-1-propenyl)-	768-49-0	891	891	0.19	C_10_H_12_	132	113562	Br
85.722	2-Propen-1-one, 1-phenyl-	768-03-6	518	618	17.49	C_9_H_8_O	132	161642	
86.131	1-Propanol, 3-chloro-, acetate	628-09-1	633	699	0.27	C_5_H_9_ClO_2_	136	3424	
87.499	Oxalic acid, isobutyl nonyl ester	-	723	826	2.50	C_15_H_28_O_4_	272	309374	
88.330	1-Hepten-4-ol	3521-91-3	648	704	1.43	C_7_H_14_O	114	1768	
88.601	non-ID				4.26				
88.876	non-ID				0.09				
89.313	2-Ethylpiperidine	1484-80-6	770	783	0.38	C_7_H_15_N	113	231734	Sk
91.945	4-Heptanol	589-55-9	687	785	1.37	C_7_H_16_O	116	1897	
93.000	1-Butanol, 4-butoxy-	4161-24-4	804	810	2.19	C_8_H_18_O_2_	146	21344	
96.432	2-Allyl-2-methyl-1,3-cyclopentanedione	26828-48-8	632	689	6.79	C_9_H_12_O_2_	152	108207	
97.644^+^	Naphthalene	91-20-3	915	919	3.98	C_10_H_8_	128	228342	F, Br, Sk, M, Sa
97.914	non-ID				4.20				
98.380	Cyclopentane, 1-isobutylidene-3-methyl-	-	664	717	0.20	C_10_H_18_	138	150621	
99.099	o-Toluic acid, 4-nitrophenyl ester	-	751	895	0.50	C_14_H_11_NO_4_	257	307460	
99.913	non-ID				1.28				
100.077	Benzoic acid, 3-amino-, methyl ester	4518-10-9	644	654	0.48	C_8_H_9_NO_2_	151	73503	
102.217	2-Butene, 2-nitro-	4812-23-1	596	690	0.06	C_4_H_7_NO_2_	101	75242	
103.393	Oxalic acid, isobutyl octyl ester	-	788	788	2.98	C_14_H_26_O_4_	258	309373	
103.528	2-Propenoic acid, 2-hydroxyethyl ester	818-61-1	483	733	0.59	C_5_H_8_O_3_	116	71287	
104.608^+^	1,9-Nonanediol	3937-56-2	769	769	0.99	C_9_H_20_O_2_	160	114694	
105.468^+^	Benzothiazole	95-16-9	827	889	0.09	C_7_H_5_NS	135	228674	Br, Sk
106.168	Ethanol, 2-phenoxy-	122-99-6	820	861	1.68	C_8_H10O_2_	138	250161	F, Br, Sk, Sa
107.094	2-Naphthalenol	135-19-3	784	867	3.69	C_10_H_8_O	144	92317	
107.739	non-ID				0.78				

The inclusion of the field sample blank aided greatly in identifying artifacts from background or impurities induced during sample transfer between different sorbent. We were able to detect a large variety of small molecular weight chemicals compounds within the WBEs consistent with the properties of VOCs ranging from small short chain hydrocarbons to long branched aliphatic and aromatics, ketones, esters, a significant amount of benzoid and halogenated compounds. All MS matching of the tentative ID peaks are supplied within the supplementary information section.

**Figure 5:**
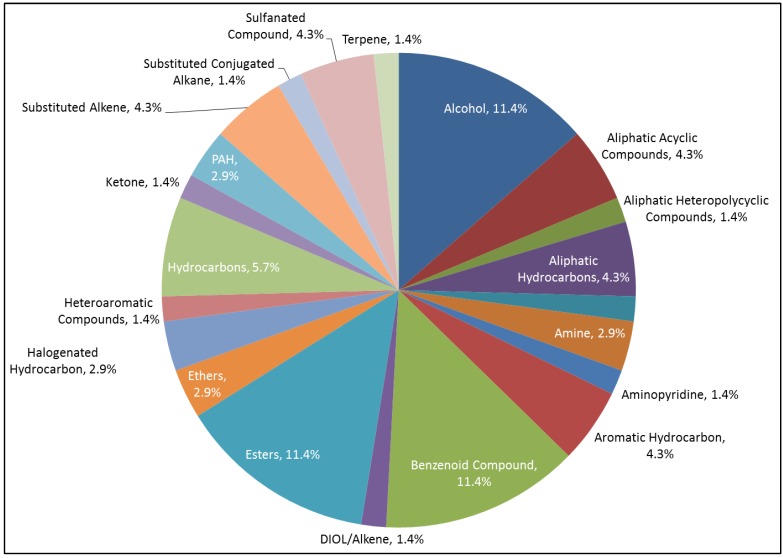
Relative abundance for chemical classes of the identified chemicals in the moribund gray whale breath exhalations (WBEs).

The relative composition of the various classes detected within the whale exhalation is visually represented in Table 1 and Figure 5. Below is a brief summary of the compounds detected across various chemical classes and tentatively matched within the exhaled whale breath. *Alcohols*: ethanol, 2-butoxy-; benzyl alcohol and 1,9-nonanediol. *Short chain aliphatic*: carbon dioxide; cyclopropane, 1,1-dimethyl-; butane, 2,3-dimethyl-; octane and pentane, 2,2-dimethyl-. *Aromatic hydrocarbons:* indane, toluene and o-xylene. *Halogenated hydrocarbons*: methane, dibromo- and benzoyl bromide. *Benzenoid compounds*: ethylbenzene; benzene, 1-methyl-4-(1-methylethyl)-; benzene, 2-ethyl-1, 3-dimethyl- and benzene, (2-methyl-1-propenyl)-. *Esters*: ethanone, 2,2-dihydroxy-1-phenyl- and 2(3H)-furanone, dihydro-4-methyl-. *Polycyclic aromatic hydrocarbons (PAH):* naphthalene. *Substituted alkane and alkenes*: 2,4,6-trimethyl-3-heptene and butane, 2-methoxy-2-methyl-. *Sulfanated compounds*: benzothiazole and carbon disulphide. *Terpenes*: limonene.

A variety of chemical compound classes were detected within the WBEs. We should mention that the necessary post-collection and transfer steps for chemical analysis needed in this pilot study may have caused some sample loss during transfer and a more extended list of detected compounds may be expected in future studies.

Of the detected compounds listed above, a large number of them have been previously reported in human clinical studies. In particular, a variety of alcohol biomarkers have been proposed in the literature [15]: 2-butoxy-ethanol; benzyl alcohol. In addition, aliphatic hydrocarbons have also been reported in humans [15]: carbon dioxide; butane, 2,3-dimethyl-; octane; and pentane, 2,2-dimethyl-. Also, aromatic hydrocarbons have been reported in humans [15]: indane, toluene and o-xylene. Similarly the halogenated hydrocarbon methane, dibromo- has been reported. The benzenoid compounds detected within this study have been reported across multiple biological matrices such a breath, skin, saliva, blood, urine and fecal matter [15]. The polycyclic aromatic hydrocarbon (PAH) Naphthalene has also been reported [15]. Additionally, the sulfanated compounds are present in humans [15]: benzothiazole and carbon disulphide. The identified terpene limonene has also been reported [15]. Furthermore, none of the esters or substituted alkane and alkenes have been found in the literature to be an endogenous compound and/or biomarkers in human breath.

In the whale breath exhalation, some of the expected VOCs that are identified in human exhaled breath such as acetone and isoprene are not found. We believe that it is due to the fact that these low mass compounds are dispersed more readily than compounds with higher molecular weight. Also, the sampling methodology was not optimized and due to the nature of the sampling technique (constrained in a river and in a moving boat) not all of the whale breath exhalation might have been collected. Therefore, we may miss sampling some of the more volatile components of the exhaled profile due to these constraints.

Certain identified compounds are rather reactive and unstable, such as *cyclopropane, 1,1-dimethyl-*, while others are not really volatile like *guanidine*. In the supplementary information, we have specified the MS matching for all compounds, and the fragmentation patterns of those specific chemical peaks are consistent with the putatively matched identification. For unambiguous chemical identification, the next step is to run chemical standards and create a MS annotation library. This is a critical procedure to ensure the utility of this method for marine mammal health monitoring.

There are two prior reports to the International Whaling Commission (IWC) of volatile organic compound analysis from whale blubber that focuses on both naturally occurring and potential contamination chemicals [53,54]. Russian aboriginal people have long observed that certain whales hunted for food have “stinky” odorous breath, and this whale meat is inedible. We compared the chemicals observed in the Klamath River whale to those reported to the IWC. Out of all of the compounds identified in our study, we found that only toluene and naphtalene are reported in the stinky gray whale’s tissue [53]. These compounds might be related to naturally derived metabolites from the gray whale as they can be present in healthy human breath, or they may be the breakdown products of other metabolites. They may also be environmental contaminants. None of the specific compounds reported in the literature for the characteristic “stinky” whale odor were found in the adult female gray whale in our study.

From our current study no health status can be provided for the moribund adult female gray whale; however, this study has shown feasibility and potential significance for future marine mammal breath biomarker discovery. For future works, if a significant amount of sample from different whales can be obtained, the analysis could be focused on levels of starvation to see if whales are metabolizing proteins from the muscles, or if there are any indications of the presence of pathogens.

VOC sampling is regarded as one of more difficult forms of sampling within analytical science. The analytes of interest are present in gaseous phase, exposed to oxidizing atmospheres and are often partially contaminated with ambient air. All of this can result in highly variable composition from sample to sample. In such cases where VOC analysis does not prove to be useful, this method of collecting WBEs still has the potential to be an incredibly important addition to the analysis of non-volatile breath biomarkers. The sampling approach is simple and relatively low cost, and has the potential to be scaled and implemented for marine mammal conservation research in the field. Other more sophisticated approaches, such as active sampling of exhale breath condensate (EBC) and non-volatile biomarkers could be possible for field trials with smaller cetaceans in capture and release exercises. However, those methods are not yet feasible for large marine mammals such as the gray whale or in field trials at sea with minimal laboratory support. Nevertheless, our approach has shown to be suitable for obtaining meaningful biogenic VOCs from gray whale breath exhalations.

## 4. Conclusions

We demonstrate that breath VOC analysis may offer a promising avenue of research for large marine mammals. The whale breath samples that we collected and analyzed displayed a large number of diverse chemical biomarker compounds across multiple classes, which could well provide an indirect means of health assessment and monitoring in these large animals. While we collected only a relatively limited number of samples in this study, the primary aims of this field trial were to: (1) assess the feasibility of collecting whale breath exhalates (WBEs) and identify any potential problems associated with this approach; and (2) assess if are there any putative biogenic VOC biomarkers present within the WBEs and, if so, assess the relative composition and abundance of these chemicals. We also note that a significant portion of the tentatively matched whale breath VOCs was also reported within the human clinical trial literature. This study is to our knowledge the first attempt to profile and characterize biogenic VOCs collected from free ranging large marine mammals such as gray whales in the field. The simplicity of our sampling approach is offset by the complexity of the post-collection and transfer steps for chemical analysis, including the transfer from the sampling bag to the Tenax^®^-TA and then to SPME for introduction to GC/MS for chemical analysis. While this worked in our pilot study, it may result in some loss of sample. However, it was necessary to make the sample stable for storage and transport back to the laboratory for subsequent analysis using our equipment. In the future, it would be better to simplify sample transfer and reduce the total number of transfer steps.

Some other suggestions for future studies include:

(1) Use a thermal desorption (TD) tube to directly introduce the breath sample into the mass spectrometer instrument with minimal sample loss. The TD tube is considered a gold standard in active VOC sampling practice, especially in outdoor environments. However, this is an expensive option as significant equipment investment in consumables and infrastructure is required. In addition to the thermal desorption unit, reusable silicone coated stainless steel tubes are also available, which include Tenax^®^-TA packing. These devices must also be conditioned. TD requires large quantity of ultra-high purity purging gas for conditioning and cleaning cycles of sorbent packing between uses. Lastly, the sorbent needs to be replaced periodically to ensure maximum absorption capacity as it is only stable for a limited period of time after cleaning, even when properly stored in optimal conditions.
(2) Use a mobile gas detection system to detect specific compounds directly from the whale blow in the field. For example, differential mobility spectrometry (DMS) is a portable sensor platform currently applied in our group to directly analyze human exhaled breath, and it represents an alternative to traditional GC/MS lab based instrument testing. However the spectral resolution of DMS is lower compared to MS, but it should still able to generate information rich spectral profiles in near real time (5–15 min) for the different chemical species present in the WBE.
(3) Once further testing is performed on exhaled cetacean breath, it should be possible to develop a mass spectral library and database for biogenic breath biomarkers from these marine mammals. This is not limited to MS alone, and other VOC sampling and analysis techniques such as DMS should be considered. It would also be useful to perform tandem MS/MS for chemical identification of the profiled breath metabolites. All of these approaches will help us to characterize unidentified biomarkers that may be associated with heath status in these important animals.
